# Supplementary material for: Real sounds influence postural stability in people with vestibular loss but not in healthy controls
Source: PLoS One. 2025 Jan 24;20(1):e0317955. doi: 10.1371/journal.pone.0317955 (PMC11760032; doi:10.1371/journal.pone.0317955)
Supplement: S1 File — (DOCX) [file pone.0317955.s003.docx]

S3. Model estimate [95 % Confidence Interval] for COP and Head AP and ML VRMS per group and sensory condition.

|  | Control Group | | | | | | Vestibular Group | | | | | |
| --- | --- | --- | --- | --- | --- | --- | --- | --- | --- | --- | --- | --- |
|  | Static Visual | | | Dynamic Visual | | | Static Visual | | | Dynamic Visual | | |
|  | No Sound | White Noise | Real Sounds | No Sound | White Noise | Real Sounds | No Sound | White Noise | Real Sounds | No Sound | White Noise | Real Sounds |
| COP ML (cm/s) | 1.27 [1.15, 1.39] | 1.29 [1.18, 1.41] | 1.26 [1.15, 1.39] | 1.45 [1.32, 1.59] | 1.47 [1.34, 1.60] | 1.44 [1.31, 1.59] | 1.46 [1.30, 1.64] | 1.40 [1.25, 1.56] | 1.48 [1.32, 1,67] | 1.70 [1.51, 1.91] | 1.65 [1.48, 1.85] | 1.66  [1.48, 1.88] |
| COP AP (cm/s) | 2.61 [2.42, 2.81] | 2.64 [2.43, 2.86] | 2.67 [2.45, 2.90] | 3.06 [2.84, 3.30] | 3.10 [2.85, 3.36] | 3.10 [2.85, 3.37] | 2.86 [2.60, 3.13] | 2.91 [2.63, 3.23] | 2.98 [2.69, 3.31] | 3.41 [3.10, 3.74] | 3.58 [3.23, 3.96] | 3.63  [3.28, 4.03] |
| HEAD ML (cm/s) | 0.79 [0.73, 0.86] | 0.79 [0.72, 0.86] | 0.78 [0.71, 0.86] | 0.93 [0.86, 1.02] | 0.94 [0.86, 1.02] | 0.93 [0.85, 1.02] | 0.95 [0.86, 1.06] | 0.91 [0.82, 1.02] | 0.97 [0.86, 1.09] | 1.10 [0.99, 1.23] | 1.14 [1.02, 1.26] | 1.15  [1.03, 1.29] |
| HEAD AP (cm/s) | 1.37 [1.28, 1.47] | 1.38 [1.28, 1.49] | 1.39 [1.29, 1.50] | 1.60 [1.49, 1.72] | 1.58 [1.47, 1.71] | 1.59 [1.47, 1.71] | 1.59 [1.46, 1.73] | 1.66 [1.51, 1.82] | 1.68 [1.52, 1.85] | 1.82 [1.67, 1.98] | 1.99 [1.81, 2.19] | 2.0  [1.81, 2.20] |
